# Environmental Geochemistry and Fractionation of Cadmium Metal in Surficial Bottom Sediments and Water of the Nile River, Egypt

**DOI:** 10.3390/toxics10050221

**Published:** 2022-04-28

**Authors:** Zozo El-Saadani, Wang Mingqi, Zhang He, Shindume Lomboleni Hamukwaya, Mahmoud S. M. Abdel Wahed, Atef Abu Khatita

**Affiliations:** 1 Earth Science and Resources Department, China University of Geoscience, Beijing 100083, China; 9101190001@cugb.edu.cn; 2 Geology Department, Faculty of Science, Zagazig University, Zagazig 44519, Egypt; 3 School of Materials Science and Technology, China University of Geosciences, Beijing 100083, China; lenihamu25@yahoo.com; 4 Geology Department, Faculty of Science, Beni-Suef University, Beni-Suef 62521, Egypt; Mahmoud.abdelwahed@science.bsu.edu.eg; 5 Department of Geology, Faculty of Science, Al-Azhar University, Nasr City 11651, Egypt; aabukhatita@taibahu.edu.sa; 6 Department of Geology, Faculty of Science, Taibah University, Al-Madinah 344, Saudi Arabia

**Keywords:** Nile river-Egypt, heavy metals, water pollution, cadmium, sediments, fractionation

## Abstract

Heavy metals such as cadmium (Cd) pollute the environment. Heavy metal pollution endangers the Nile River since it serves as an irrigation and freshwater source for the cities and farms that line its banks. Water and sediment samples from the Nile River were tested for Cd content. In addition, a sequential experiment analytical method was performed to determine the metal’s relative mobility. According to the data, there is an average of 0.16 mg kg^−1^ of Cd in sediments. The BeniSuef water treatment plant and brick factory, the iron and steel factory of Helwan, the oil and detergent factory of Sohag, and the discharge of the cement factory in Samalut had the greatest concentration of Cd in their vicinity. According to the risk assessment code, there are four categories of Cd: residual (57.91%), acid-soluble (27.11%), reducible (11.84%), and oxidizable (3.14%). Bioavailable and mobile Cd levels in sediment and water were found in Beni Suef, Aswan; Helwan; Samalut; Sohag; and Helwan. Because the other metal is highly bioavailable, its concentration is not a risk factor at the Samalut station. Cd’s toxicity and bioaccumulation make it an extra hazard to aquatic animals and human life. There should be a deterministic approach to monitoring Cd near industrial sources.

## 1. Introduction

Once heavy metals are discharged into the environment (air, soil, water, and sediments), they don’t disappear; sediments, soil, and biota absorb them. As a result, sediments, water, and biota play a key role in determining the extent of environmental toxicity of dangerous compounds [1,2,3]. It is widely accepted that certain elements are essential for life on earth, such as iron, copper, zinc, and manganese. Heavy metals like mercury, lead, cadmium, and others are not necessary for life, but they can be harmful even at deficient levels [4]. Human health can be negatively impacted regardless of exposure to high or low levels of these pollutants through the air, water, or food (plants and animals). Sediment geochemical studies can understand Cd pollution’s properties, distribution, and causes. Cadmium is a transition metal with a density of 8.642 g cm^−1^ and a molecular weight of 112.40 g mol^−1^. It is found as a minor constituent in mineral sulfides, especially zinc sulfides such as Sphalerite and Wurtzite; hence, its natural sources from the earth’s crust include volcanic eruptions and the weathering of rocks containing Cd [5,6]. Volcanoes, airborne soil particles, biogenic materials, sea spray, and forest fires all contribute to the release of cd into the atmosphere. Cd sources include cement manufacturing, mining, and manufacture of non-ferrous metals, iron and steel production, coal combustion, waste incineration, municipal wastes, and the application of mineral fertilizers. Sediment from rivers and lakes contains Cd concentrations of up to 5 mg/kg, whereas marine sediments include concentrations of between 0.03 and 1 mg kg^−1^ of metal [7].

According to the Environmental Protection Agency (EPA) study, the Cd average ranges from 5 to 20 ng L^−1^ in open seawater [8]. Acetate, chloride, and sulfate are the most water-soluble inorganic cadmium-based compounds; nevertheless, insoluble oxides, carbonates, and sulfides are impossible to remove from the environment (e.g., soil) [9,10]. The Cd levels in European agricultural soils ranged from 0.06 to 0.6 mg kg^−1^ [11]. The kidneys of cattle, poultry, and pigs contained Cd concentrations ranging from 0.01 to 0.50 mg kg^−1^ [12]. Paintbrushes washed under the tap can spread roughly 110 kg of Cd to agricultural soil each year [13]. The typical human consumption of Cd is 1.5 g kg^−1^ of body weight (1.8 g for vegetarians), which can be calculated based on the Cd content of specific foods [14]. The daily intake of Cd is increased by 2 to 4 g by smoking one package of cigarettes [15]. Cd poisoning can lead to high doses of hypercalciuria, kidney stones, lung cancer, and prostate cancer [8]. Metal content in sediments is crucial in regulating metal bioavailability to river organisms [16]. Cd is a hazardous heavy metal with long-term health and environmental consequences even at low exposure levels. The two states of cadmium oxidation are metallic (rare; insoluble in water) and divalent (Cd^+2^) (predominant and soluble in water). The Free Cd^+2^ ion is the main toxic form of Cd; however other forms of cadmium, for example, those bound to various ligands, may also cause adverse effects. The toxicity, bio-accumulative potential, and non-biodegradability of cadmium-based content were monitored in Egypt’s Nile River to determine the consequences on aquatic, animal, and human health. This study aimed to analyze the current concentrations of Cd in Nile waters and sediments, illustrating its distribution and potential sources, determining the degree of contamination, and how much Cd is bioavailable. As a result, this study will help better understand the current state of the environmental impact of heavy metals along the Nile River.

## 2. Materials and Methods

### 2.1. Study Area

A total of 11 African countries, including Egypt, share borders with the Nile River, which covers a distance of 6650 km and flows into the Mediterranean Sea. For decades, this river has been a vital primary source of fresh water for humans and animals and a source of irrigation for the dry country around it. Today, the river still provides irrigation and serves as a vital transit and trading route. At the same time, toxic substances are being discharged into the river. The White, Blue, and Atbara Nile Rivers entered the main Nile. Arabian–Nubian Shield Basement rocks, Phanerozoic sedimentary cover, Ethiopian Highlands (basalt), and aeolian sources from the highlands of the Red Sea of Egypt supply sediments to the Nile’s trunk [17,18,19]. The Nile River provides 80 to 85% of water for the agricultural sector and 65% of the water needed for industrial purposes, and it receives over 57% of the effluents generated [20]. The Nile receives massive amounts of agricultural effluent, which contains a variety of chemical contaminants related to the common use of fertilizers and pesticides. Significant Cd pollution in the Nile River bottom sediments between Aswan and Esna, near the phosphate shipping harbors [21]. The Nile River and its tributaries are pretentious by various human-caused activities, including the disposal of sewage sludge and wastewater, agricultural activities, industrial processes, and the use of phosphate fertilizer [2,22,23,24]. According to Egypt’s Nile River studies [25,26,27,28,29,30], hazardous metals such as Cd, Pb, and Fe have been found in important economic fish species, aquatic plants, and water. Increasing pollution and dwindling Nile water levels are Egypt’s most pressing issues, especially regarding the completion of the new dam construction project.

### 2.2. Sampling and Geochemical Analysis

**Figure 1:**
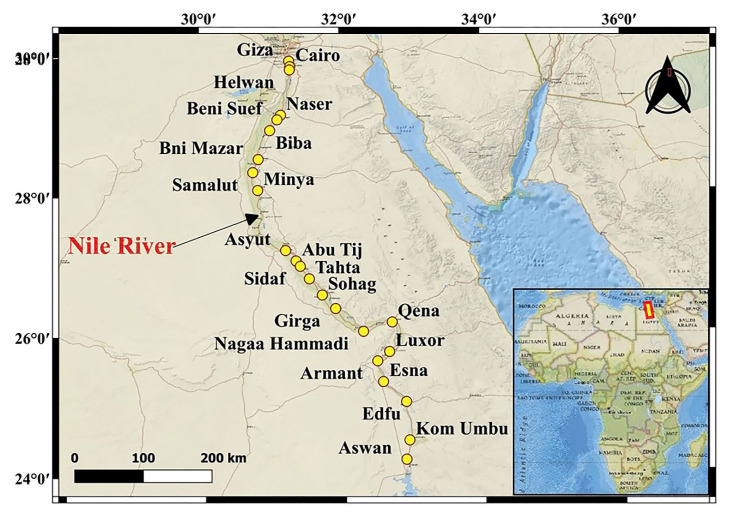
The location map of studied samples along Nile River, Egypt.

In September 2019, 23 representative sediment and water samples (from two banks and the middle) were carefully selected from Aswan to Cairo (Figure 1) to evaluate Cd concentration and fractionation in the bottom sediments and determine the anthropogenic sources of pollution along the river. A grab sampler (Ekman type) was used to capture the sediments rinsed between sites with distilled water. In an oven at 70 °C, the sediments were dried for around 26 h before being kept for chemical testing. A GPS tracker was utilized to locate the sampling locations’ latitude and longitude and their elevations. This method of analyzing the total Cd content in sediments uses a chemical reaction involving the digestion and addition of HCl, HNO_3_, and 2 mL HF to 0.25 g of dry sediment. Finally, the digested solutions were subjected to inductively coupled plasma mass spectrometry (ICP-MS) (Agilent 7900, USA) and inductively coupled plasma atomic emission spectroscopy (ICP-AES) (Agilent 5110, Santa Clara, CA, USA) analysis at ALS CEMEX (Guangzhou, China) Co., Ltd-China, respectively. To monitor the state of the equipment and ensure quality, a reference solution was measured after every five samples were analyzed. Every chemical reagent utilized was of analytical grade.

Using a waterproof (PH/EC/TDS) and portable temperature meter, the pH, temperature, and total dissolved solids (TDS) of water samples were evaluated simultaneously with the collection of water samples using a portable meter of (HI98129.HI98130, HANNA, Rhode Island, WA, USA). Before the experiment, the PH meters were calibrated with standard solutions. A professional waterproof portable PH/ORP Meter (HI98190, HANNA, Rhode Island, WA, USA) was used to determine the oxidation-reduction potential (ORP). All samples were acidified with ultrapure HNO_3_ acid in a 30 mL LDPE bottle washed with ultrapure water and 10% HNO_3_ acid. Both the acid and the water used were of the highest quality. Temperature-controlled storage was employed for storing water samples at a temperature (4 °C) before analysis, as per standard procedures [31]. ICP-MS was used to determine the amount of Cd in the water samples.

The laser diffraction method was used for grain size analysis on representative samples of sediments prepared [32]. Laser diffraction became the standard method for sediment particle size measurement [33,34,35]. Analysis was performed with an alight scattering apparatus (Winner 2308A, Jinan, China) equipped with a >3 mW Helium-Neon laser with a wavelength of 632.8 nm. The beam wavelength of 2.4 mm operates from 0.1 to 2000 µm (Table 1).

### 2.3. Sequential Extraction Fraction Method

**Figure 2:**
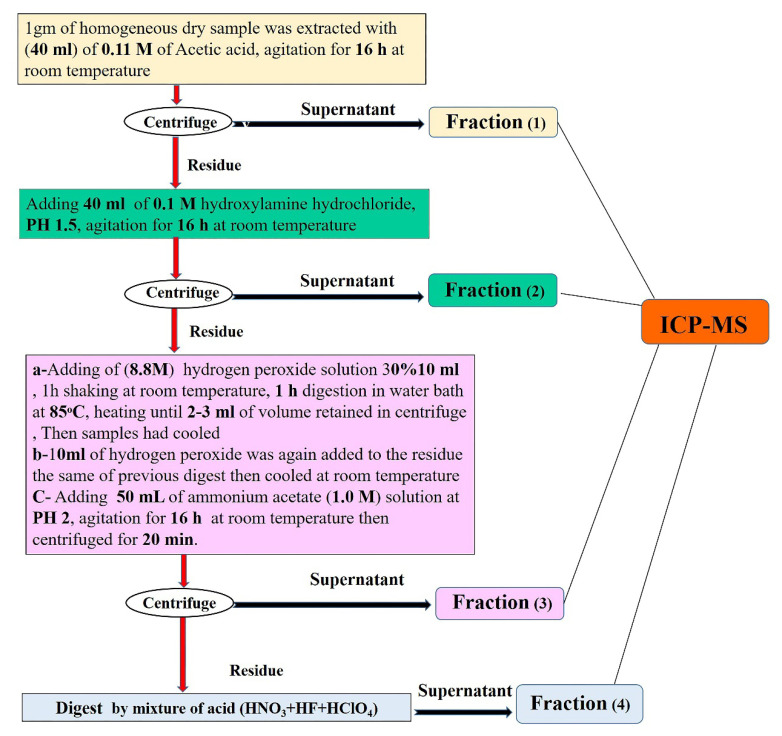
Flowchart of sequential extraction procedures.

In soils and sediments, single extractions are utilized to rapidly evaluate the exchangeable metal fraction [36,37,38]. However, there are a variety of trace element speciation procedures that have environmental implications in soils and sediments [39,40,41]. For the chemical separation of Cd in sediments, the European Community Bureau of Reference (BCR) sequential extraction procedure was recommended [42,43]. BCR procedure has been widely used to detect specific chemical forms of heavy metals in various environmental mediums, including sediments. The BCR-701 sediment certified reference material was used to validate it, which included certified and indicated extractable amounts of Cd, Ni, Cu, Pb, Cr, and Zn [44]. Many specialists used and approved this method [45,46,47,48,49,50,51]. Before the BCR process, the sediments were utterly dried in an oven at 40 °C for around 48 h. A shaker was used to mix the sediments at room temperature for 16 h. To get the fractions, each step’s fraction extraction was centrifuged at 3000 rpm for 20 min and then placed in a polyethylene centrifuge tube. A 20-min centrifuge was performed, followed by a 15-min automated shaker wash at 3000 rpm for the residue. The supernatant was decanted, leaving a residue. This separation took place in the geochemical laboratory of the China University of Geo-science, Beijing. Each sample was cleaned with 10 mL of ultrapure water before and after extracting the data. After soaking in dilute HNO_3_ overnight, all polypropylene and glassware were washed with ultrapure water before use [51]. The sample’s residues were digested with a mixture of acids (HNO_3_ + HF + HClO_4_) [52]. There was no question about the quality of the reagents and the standard solutions utilized in this experiment. Every fraction’s metal content was measured using ICP-MS. A schematic representation of the extraction procedure is provided in a flowchart (Figure 2).

### 2.4. The Pollution Level Estimation

The distribution of metal concentrations in sediments and comparison with non-polluted backgrounds are necessary to determine the mechanisms of geochemical distribution and accumulation of heavy metals and provide essential information for assessing environmental health risks in aquatic systems. Assessing the quantity of Cd in the environment and the potential for ecological risk requires the use of environmental pollution indices such as the Enrichment factor (EF), Contamination factor (CF), Geo-accumulation index (I_geo_), and Ecological potential risk (Er) [53,54]. The contribution of anthropogenic sources normalized to the metal concentration background value of the upper continental crust [55] is as follows:

#### 2.4.1. Enrichment Factor (EF)

To determine the contribution of anthropogenic sources to the natural levels of heavy metals in the Nile River sediments, enrichment factors for heavy metals in sediments are determined. The comparable upper continental crust values [55] were employed as a background in our scenario. The Enrichment Factor (EF) of Cd was calculated using the formula below.

(1) $$\mathrm{EF}=\frac{C_{i}/C_{r}}{B_{i}/B_{r}}$$

where *C_i_* and *C_r_* are the concentrations of the metal and the reference metal in the sample (Al), while B_i_ and B_r_ are the background concentrations of the metal and the reference (Immobile elements such as Al have been used as the background metals [56] for EF calculation in this study. According to Pereira et al., EF can be classified as follows:

EF < 2 indicates no or minimal enrichment, EF between 2 and 5 indicates moderate enrichment, EF between 5 and 20 indicates significant enrichment, EF between 20 and 40 indicates very high enrichment, and EF > 40 indicates extreme enrichment [57,58].

#### 2.4.2. Contamination Factor (CF)

CF can represent the level of contamination; it is a useful tool for monitoring contamination in sediments over time. It is calculated using the following formula:

(2) $$\mathrm{CF}=\frac{C_{\mathrm{metal}}}{C_{\mathrm{background}}}$$

*C_metal_* is the metal concentration, and *C_background_* is the background value of UCC [55]. The contamination degrees are categorized according to their values as follows CF < 1 = low contamination, CF = (1 − 3) is moderate contamination, CF = (3 − 6) is considerable contamination, and CF > 6 = very high contamination [59].

#### 2.4.3. Index of Geo-Accumulation (I_geo_)

An indicator called geo-accumulation index was initially defined by Müller [60], the first to use the term I_geo_. To measure the extent to which anthropogenic pollution, geochemical background value, and natural diagenesis enrichment. To determine the I_geo_, the following equation was used:

(3) $$I_{\mathrm{geo}}=\log_{2}\left(\frac{C_{n}}{1.5\;B_{n}}\right)$$

where C_n_ is the measured content of an element a (_n_), B_n_ is the geochemical background of element n [55], and a constant of 1.5 is used due to metal fluctuations in the soil as well as some minimal anthropogenic influences [59]. I_geo_ values are classified as follows: I_geo_ < 0 unpolluted, I_geo_ (0–1) unpolluted to moderately I_geo_ (1–2), moderately polluted I_geo_ (2–3), moderately to heavy polluted I_geo_ (3–4), heavy polluted I_geo_ (4–5), heavy to extreme polluted and I_geo_ > 5, is extremely polluted [60].

#### 2.4.4. Ecological Risk Index (*Er*)

This index assesses the potential risk to the ecology of one or more constituents [61]. When the prospective ecological risk factor and the toxicity response coefficient were taken into account, *Er* reflected the sensitivity of the biological community. The *Er* is calculated as follows:

(4) $$E_{r}=C_{f}^{i}*T_{r}^{i}$$

where $C_{f}^{i}$ is the contamination factor, $T_{r}^{i}$ is the toxicity response coefficient of each element (Cd = 30) [61,62] and *Er* is the ecological risk factor of each element [63]. *Er* values were categorized as follows *Er* <40 is low pollution, 40 < *Er* < 80 moderate potential risk, 80 < *Er* < 160 high potential risk, 160 < *Er* < 320 very high potential risk, and *Er* > 320 dangerous [57].

## 3. Results

### 3.1. Cd Distribution in Sediments

**Figure 3:**
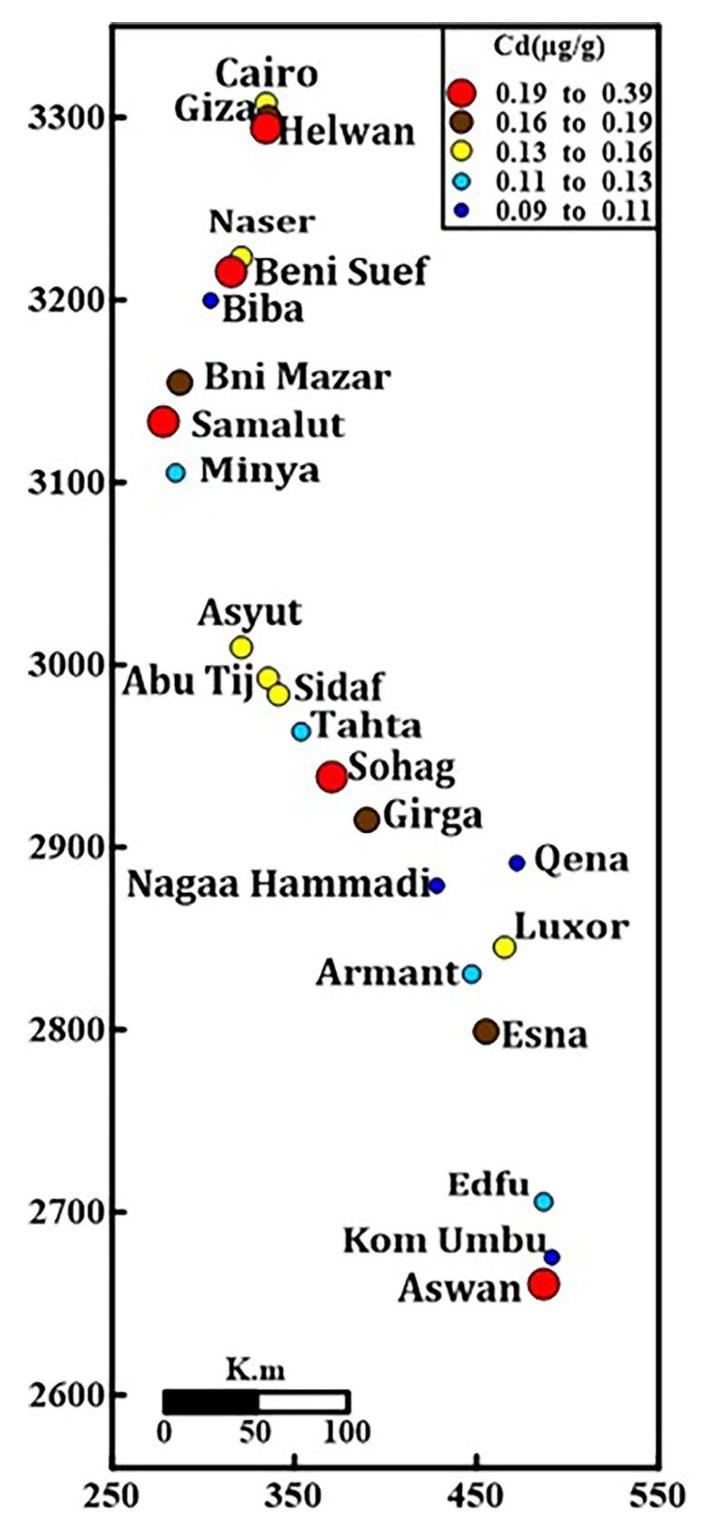
Symbol map of Cd concentration (mg kg^−1^) of the Nile River mainstream sediments.

The average particle size analysis for fine sand, silt, and clay was 81.54%; 15%; 2.47%, respectively; this indicates that the High Dam effect and low weathering have resulted in less clay concentration. From 0.09 to 0.38 mg kg^−1^, the Nile River bottom sediments contain Cd, with an average value of 0.16 mgkg^−1^ (Table 1). Benisuef (0.38 mg kg^−1^), Aswan (0.27 mg kg^−1^), Helwan (0.23 mg kg^−1^), Samalut (0.2 mg kg^−1^), and Sohag (0.19 mg kg^−1^) had the most significant concentrations (Figure 3). In comparison, the average of Cd in this investigation and the Rosetta branch (0.8 mg kg^−1^) [64] shows that the increase from upstream to downstream (South to North) is related to the increase in industrial activities, as quoted by Abou El-Anwar et al. (2021). On the other hand, the Cd average is higher than that of Nile sediments in the Sohag governorate (0.004 mg kg^−1^) [65] and of the Cairo sector (0.06 mg kg^−1^) [66], while less than that of the Assuit governorate (0.6 mg kg^−1^) [23] and Nasser Lake (0.183 mg kg^−1^) [67]. Comparatively, with worldwide rivers and backgrounds, the mean value of Cd in the current study is more than that of UCC [54] while less than that of world rivers (1.4 mg kg^−1^) [68] and USEPA (0.61 mg kg^−1^) [69] (Table 2). There is no significant correlation between Cd and (sand, silt, and clay percent) (Table 3). The anthropogenic source is supported by the negative correlation of Cd with Zr (−0.15) (Table 3) because Zr has been commonly employed in geochemical investigations of mineral weathering as a conservative lithogenic element [70,71].

### 3.2. Pollution Level

**Figure 4:**
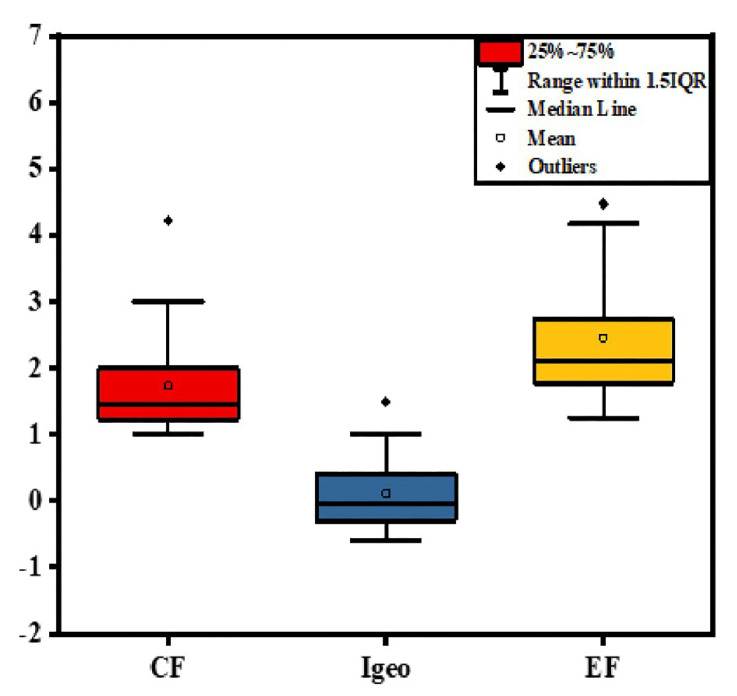
Box plot of Contamination factor (CF), geo-accumulation index (I_geo_), and enrichment factor (EF) according to (McLennan, 2001) Nile River sediments.

**Figure 5:**
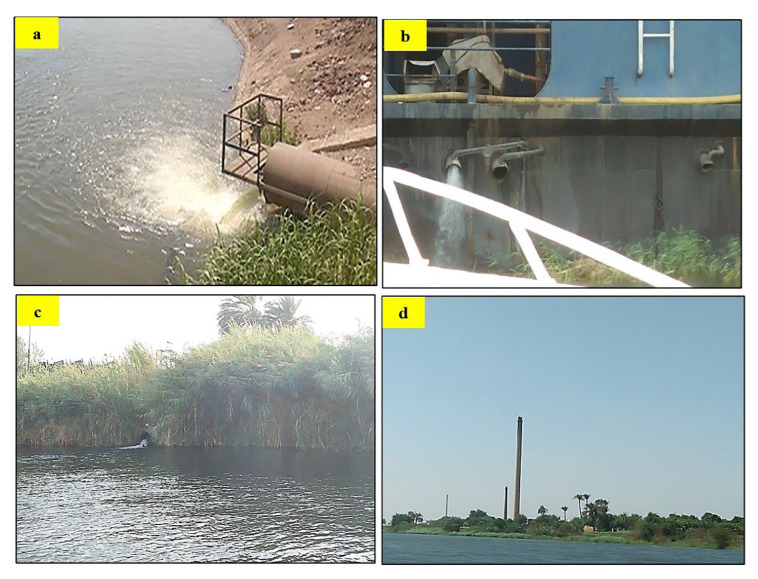
Point anthropogenic sources; (**a**) discharge of sugar refining factory, Giza in Nile River, (**b**) Cruise discharge in the Nile, Sohag, (**c**) Agriculture discharge in Nile, Luxor, and (**d**): Brick factories, Beni Suef on Nile Bank.

Heavy metal pollution has become incredibly critical [77]. All pollution indices were calculated related to UCC [55] presented in Table 1. The mean value of the EF was 2.46, with a range (1.25–5.88) indicating low to moderate enrichment. Furthermore, the CF average of Cd is 1.74 with a range of 1–4.22, showing moderate to high contamination (Table 1). Although, the I_geo_ average is 0.12 with a range of −0.58–1.49, depicting that the Nile River sediment is unpolluted to moderately polluted with cadmium (Table 1 and Figure 4). The ecological potential risk index ranged from 30 to 126.67, with an average of 52.17, indicating a low to high risk of cadmium (Table 1). Beni Suef, followed by Aswan, Helwan, Samalut, and Sohag samples, recorded the highest value of pollution degree. The difference in cadmium concentration and pollution level along the river may be related to the near and far from the anthropogenic source of Cd mobility and discharge points (Figure 5). Cd is one of the banned elements regarded as the most toxic to aquatic life and people; increased exposure produces both noncarcinogen and carcinogen dangers such as renal illness, bone damage, and even cancer [78].

### 3.3. Sequential Extraction Fractions of Cadmium

**Figure 6:**
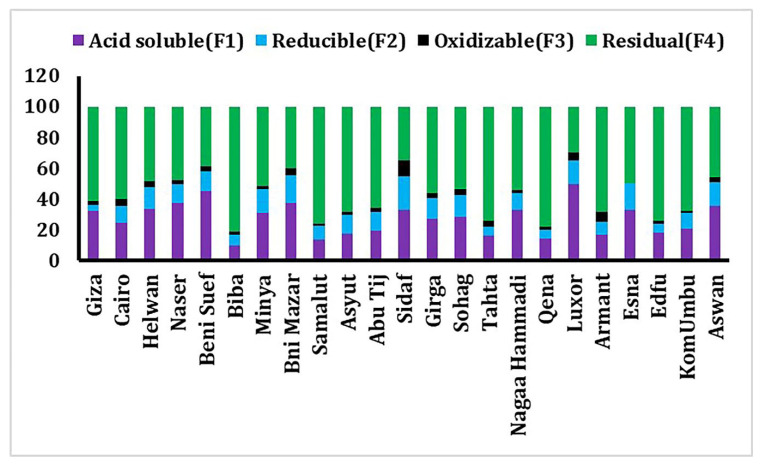
Results of Cd BCR sequential extraction fractions (%) and relative abundance in each location of sediment samples.

**Figure 7:**
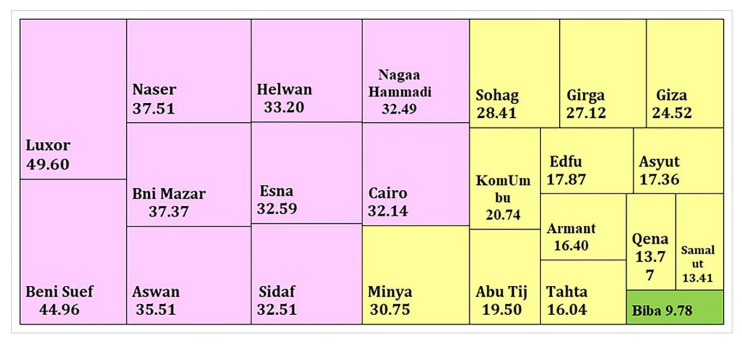
Treemap of cadmium metal potential Risk assessment Code (RAC) from all study positions to Nile River mainstream sediments.

Cd can harm human health and the environment, even at low doses. Air pollution, tobacco smoke inhalation, and tainted food expose humans to Cd [79]. Exchangeable and carbonate, Fe-Mn oxyhydroxide (reducible), organically bound (oxidizable), and residual geochemical forms are important for determining the biological form of cadmium as well as the solubility, mobility, and toxicity of metals bound to various sediment phases [80]. Metals attached to the metals bound to the exchangeable fraction are easily accessible, but those in the carbonate phases are more mobile with increasing acidity [39,51]. The residual fraction is considered to represent the unreactive phase. The cadmium fractions follow this order: residual (57.91%) > Acid soluble (27.11%) > Reducible (11.84%) > oxidizable (3.14%) (Figure 6 and Table 1). Cadmium was mostly concentrated in the residual fraction >74% at Biba, Tahta, Samalut, Edfu, and Qena. In reducible, a portion of the Cd fraction may form stable complexes with Fe and Mn oxides [81]. Cd positively correlated with F1 fraction (r = 0.5) (Table 3). The risk assessment code (RAC) was suggested for assessing the availability and environmental risk of heavy metals [82,83]. RAC is applied to the bioavailable speciation acid-soluble fraction in this investigation. If metal content in this fraction (acid-soluble) is less than 1% of the total, it is deemed safer for the environment; the range of 1–10% is low risk, 11–30 is medium risk, and 31–50 is a high risk, and 50–100% is very high risk. So, the station’s samples are from medium to high risk, apart from Biba being at the lowest risk. The high risk was recorded at (Luxor, Beni Suef, Nasser, Bni Mazar, Aswan. Helwan, Esna, Sidaf, Nagaa Hammadi, and Cairo) were >31% (Figure 7), indicating high bioavailability and mobility at these stations. In this investigation, all stations represent the high risk, medium risk, and low risk represent (43%, 52%, and 5%, respectively). This medium-high risk of Cd makes it easy to enter the food chain. The toxicity of Cd to aquatic organisms is related to the availability of free ionic concentration. Animals and the human body through the food chain are impacted by the high concentration of heavy metals [84]. In correlation with the bioavailability of worldwide rivers, the cadmium bioavailability in this study is moderate and poses a risk to the environment (Table 4).

### 3.4. Multivariate Statistical Analysis (Cluster Analysis)

**Figure 8:**
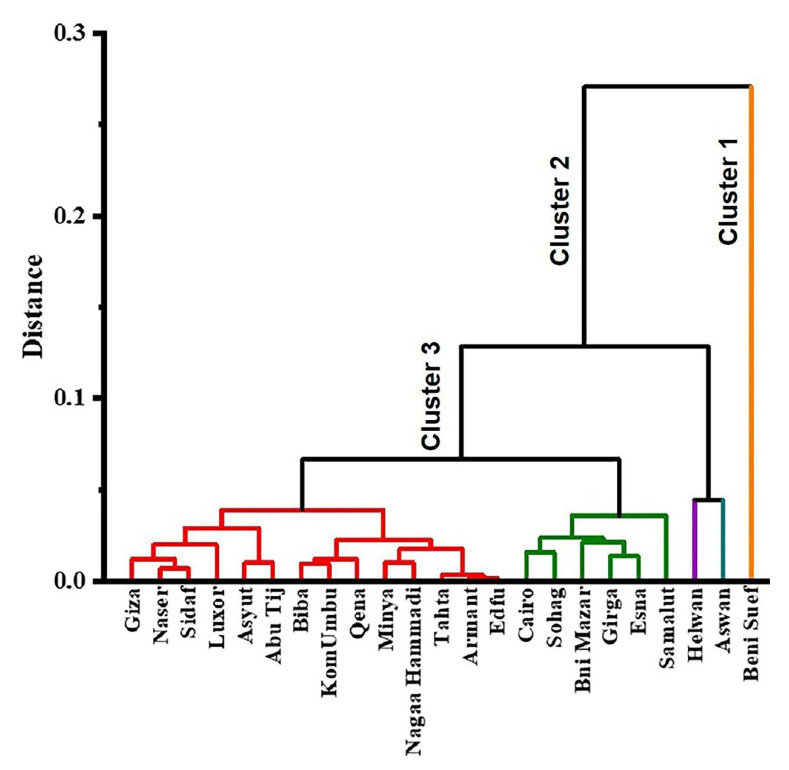
Dendrogram: a cluster of variables based on similarity.

Cd metal contamination in ecosystems needs to be identified and evaluated while considering both natural and artificial influences. Cd concentrations in sediments and water with a RAC were used as variables in a cluster analysis throughout the Nile River’s mainstream. The cluster analysis (Figure 8) shows three sources of Cd at all stations: Beni Suef is the only sampling site in Cluster 1 that is located near agricultural discharge and industrial activities (water treatment plant, brick factory). Cluster 2 comprises two sampling sites (Helwan and Aswan) close to manufacturing activities (iron and steel mills and a sugar refinery). Cluster 3 consists of 20 sampling sites (Cairo, Sohag, Bni Mazar, Girga, Esna, Samalut, Giza, Naser, Sidaf, Luxor, Asyut, AbuTij, Biba, KomUmbu, Qena, Minya, Nagaa Hammadi, Tahta, Armant, and Edfu) near bridges, dams, water treatment plants, sugar production plants [51], and agricultural expulsion facilities are the most common locations.

### 3.5. Analysis of Cadmium Concentrations in Water

Agricultural, industrial, household, and touristic activities along the Nile’s banks affect the river’s water quality upstream to downstream [91]. Water pollution is caused by population increase, urbanization, and industrialization, where waste from industrial, agricultural, and residential activities is discharged into rivers worldwide [92,93]. Aquatic and terrestrial organisms bioaccumulate cadmium, but it is toxic to aquatic organisms at low concentrations [94]. In this paper, the median Cd concentration in water is 4 µg/L (0.004 mg/L) (Table 5). The high cadmium concentration in water was recorded at Cairo, Giza, Helwan, Beni Suef, Sohag, Qena, and Samalut with values (0.009, 0.01, 0.008, 0.007, 0.007, 0.006, and 0.006 mg/L, respectively) more than standard limits [95]. Unpolluted natural waters are usually below1 µg/L [96]. Furthermore, the Cd average in water according to EPA is 3 µg/L [97], WHO is 5 µg/L [95], and CCME is 0.18 µg/L [98]. In comparison, the current study Cd average is more than recorded from Aswan to Beni Suef (1 µg/L) and (3.5 µg/L) [99] and [25], respectively, while it less than from Aswan to Delta was (5.9 µg/L) [100] because of significant pollution at Delta. The solubility toxicity of chemicals and heavy metals can be affected by the PH of the Water; the solubility of heavy metals occurs at low PH [101]. Most marine animals favor a pH range of 6.5–9.0. As hydrogen ions rise, metal cations such as lead, aluminum, cadmium, and copper are released into the water rather than absorbed by the sediment, causing heavy metal concentrations to rise and their toxicity to increase. So, cadmium is negatively correlated with PH (−0.35) (Table 3). Recorded PH ranged from 7.9–9 with a median (8.4); however, PH according to EPA is 6.5–8.5 [97] and WHO is 6.5–8 [95], and Egyptian regulation is 7–8.5. PH 9 is the highest recorded value at Qena. According to Niyogi et al., low PH may protect fish against acute Cd toxicity. Oxidation-reduction potential (ORP) determines a substance’s capability to either oxidize or reduce another substance and denotes how sanitized or contaminated water is based on its oxidation and reduction properties [102]. ORP is negative when your sample is at quite a low redox level but positive at the oxidic level. The ORP average (345.87 mV) is lower than the WHO limit value (700 mV) [95]. The average temperature was (28.42 °C), and the average TDS was 158.39 mg kg^−1^, lower than the Egyptian regulatory and EPA [97] (500 mg kg^−1^) limits.

## 4. Discussion

Earthworms, poultry, horses, cattle, and animals have been found to have high amounts of cadmium bioaccumulation [94]. Cd is a non-essential metal progressively absorbed by humans and more mobile than most heavy metals in aquatic environments. Algae and suspension feeders absorb dissolved cadmium in the aquatic environment; fish are more likely to absorb cadmium in freshwater [94]. Cadmium concentration differences along the river with average from Aswan to Cairo is (0.16 mg kg^−1^) and is recorded high concentration and pollution degree near the water treatment plant and brick factory of BeniSuef, the iron and steel factory of Helwan, the oil and detergent factory of Sohag, and discharge of cement factory in Samalut (Table 1). A negative correlation with Zr has shown its anthropogenic source (Table 3). Due to the increase in population growth, urbanization, and industrialization along the river, the Cd was higher than in previous studies conducted on Egypt’s Nile River. Corresponding to the risk assessment code [103], Cd is high risk at Luxor, Beni Suef, Nasser, BniMazar, and Aswan.

**Figure 9:**
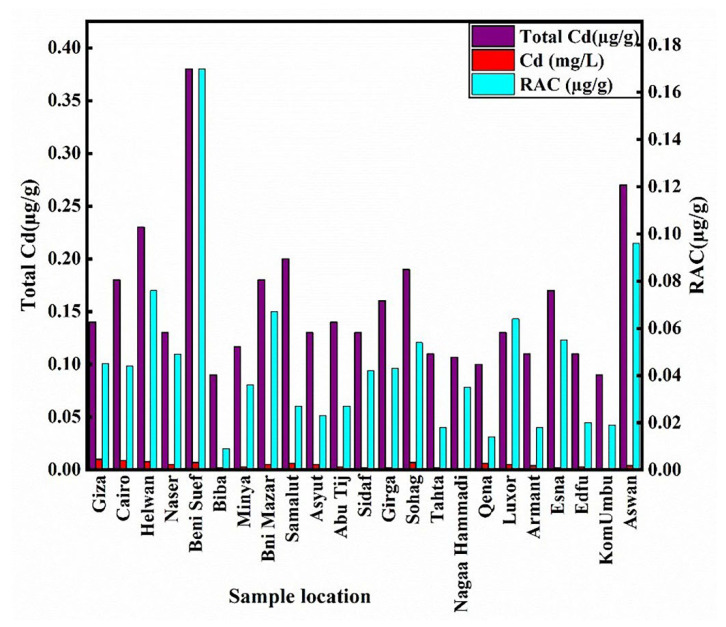
Relation between Concentration of Cd in sediments (mg kg^−1^) mg kg^−1^ and Water mg L^−1^ with risk assessment code (RAC) (mg kg^−1^) of samples along Nile River^-^ mainstream.

Moreover, water cadmium concentrations are higher than permissible limits in Cairo, Giza, Helwan, Beni Suef, Sohag, Qena, and Samalut (Table 5). Cluster analysis reveals three pollution sources: agriculture discharge, industrial activities, and (domestic and sewage sludge). The Cd concentration is significant at Beni Suef, Aswan, Helwan, Samalut, and Sohag in sediments and water with high bioavailability and mobility (Figure 9) related to the vicinity of anthropogenic sources (Figure 5). At the same time, the others with low content have high bioavailability, so the concentration is not the risk indicator of any metal. Some stations along the Nile River have recorded high content of Cl^−^ and SO_4_^2−^ [101], so the probability of cadmium soluble compounds such as chloride and sulfate may be formed. The toxicity increases, so the cadmium pollution in water and sediments in these stations may affect fish and then humans. Contaminated food is the most toxic source of cadmium to humans. It is greatly enhanced in persons who regularly eat shellfish and fish organ meats (liver and kidney) [94]. We recommended more research on aquatic organisms and humans, especially in these locations. Environmental lawyers and legislators must develop regulations to ensure water is managed correctly for the identified uses.

## 5. Conclusions

Heavy metal pollution endangers the Nile River since it serves as an irrigation and freshwater source for the cities and farms that line its banks. Cd pollutes the environment and is toxic at low concentrations. The cadmium average in sediments is (0.16 mg kg^−1^). The most significant concentrations were recorded at Benisuef (0.38 mg kg^−1^), Aswan (0.27 mg kg^−1^), Helwan (0.23 mg kg^−1^), Samalut (0.2 mg kg^−1^), and Sohag (0.19 mg kg^−1^). The pollution level of cadmium in sediments is moderate to high at all sample stations along the river. The concentration and distribution of Cd in rivers are affected by the vicinity of anthropogenic sources such as household waste, sewage sludge, agricultural runoff, and industrial activity. The Cd fractions follow this descending order: residual (57.91%), acid-soluble (27.11%), reducible (11.84%), and oxidizable (3.14%). The high cadmium concentration in water was recorded at Cairo, Giza, Helwan, Beni Suef, Sohag, Qena, and Samalut with values (0.009, 0.01, 0.008, 0.007, 0.007, 0.006, and 0.006 mg L^−1^, respectively) more than standard limits. Beni Suef, Aswan, Helwan, Samalut, and Sohag all have significant bioavailability and mobility of Cd in sediment and high content in water. Accordingly, the river’s contamination must be thoroughly investigated, particularly in the vicinity of industrial points of origin in the areas stated. The primary effects of Cd on the environment and human health can be summarized as ecosystem contamination and exposure-related health issues. Egypt’s high Cd concentration could become a problem if it is not carefully managed. We argue for continuing studies on aquatic organisms and humans in these places.

## Figures and Tables

**Table 1:** The concentration of Cd (mgkg^−1^), calculated Pollution indices (CF, Er, I_geo_, and EF), and fractions distributions (%) of Nile River sediments.

Fractions at Sediments %												
Sample Location	Cd	CF	Er	I_geo_	EF	Sand%	Silt%	Clay%	(F1)	(F2)	(F3)	(F4)
Giza	0.14	1.56	46.67	0.05	2.17	85.6	12.1	2.3	32.14	3.73	2.37	61.76
Cairo	0.18	2.00	60.00	0.42	2.76	85.6	13.2	1.2	24.52	10.59	4.80	60.09
Helwan	0.23	2.56	76.67	0.77	3.78	53.4	41.1	5.5	33.20	14.20	3.88	48.72
Naser	0.13	1.44	43.33	−0.05	1.96	96.4	3.3	0.3	37.51	11.85	2.65	47.99
Beni Suef	0.38	4.22	126.67	1.49	5.88	67.9	28.9	3.2	44.96	12.95	3.34	38.74
Biba	0.09	1.00	30.00	−0.58	1.44	94.4	5.3	0.3	9.78	6.86	1.65	81.72
Minya	0.12	1.33	40.00	−0.17	1.88	81.8	15.7	2.5	30.75	15.56	1.92	51.77
Bni Mazar	0.18	2.00	60.00	0.42	2.48	51.6	45.2	3.2	37.37	17.90	4.37	40.36
Samalut	0.20	2.22	66.67	0.57	4.17	95.9	3.8	0.3	13.41	8.97	1.60	76.02
Asyut	0.13	1.44	43.33	−0.05	2.18	85.1	13.1	1.8	17.36	12.04	2.08	68.52
Abu Tij	0.14	1.56	46.67	0.05	2.04	95.8	3.8	0.4	19.50	12.13	2.44	65.94
Sidaf	0.13	1.44	43.33	−0.05	1.71	40.1	51.7	8.2	32.51	22.37	9.96	35.15
Girga	0.16	1.78	53.33	0.25	2.11	68.5	28.3	3.2	27.12	13.23	3.02	56.64
Sohag	0.19	2.11	63.33	0.49	2.75	74.7	21.9	3.4	28.41	13.79	3.85	53.95
Tahta	0.11	1.22	36.67	−0.30	1.78	71.5	25.3	3.2	16.04	5.97	3.68	74.31
Nagaa Hammadi	0.11	1.22	36.67	−0.30	1.87	83.3	14.6	2.1	32.49	11.49	1.32	54.70
Qena	0.10	1.11	33.33	−0.43	1.54	98.1	1.6	0.3	13.77	6.17	1.60	78.46
Luxor	0.13	1.44	43.33	−0.05	1.96	90.6	7.8	1.6	49.60	15.36	5.15	29.89
Armant	0.11	1.22	36.67	−0.30	1.68	88.6	8.8	2.6	16.40	8.82	6.01	68.78
Esna	0.17	1.89	56.67	0.33	2.22	98.7	1.1	0.2	32.59	17.40	0.21	49.80
Edfu	0.11	1.22	36.67	−0.30	2.43	89.6	1.3	9.1	17.87	5.70	1.74	74.69
KomUmbu	0.09	1.00	30.00	−0.58	1.25	93.3	6.1	0.6	20.74	9.92	1.14	68.19
Aswan	0.27	3.00	90.00	1.00	4.47	85	13.6	1.4	35.51	15.28	3.39	45.82
**Average**	0.16	1.74	52.17	0.12	2.46	81.54	15.98	2.47	27.11	11.84	3.14	57.91
**Maximum**	0.38	4.22	126.67	1.49	5.88	98.70	51.70	9.10	49.60	22.37	9.96	81.72
**Minimum**	0.09	1.00	30.00	−0.58	1.25	40.10	1.10	0.20	9.78	3.73	0.21	29.89

**CF**: Contamination factor; **Er**: Ecological potential risk, **I_geo_**: Geo-accumulation inde, and **EF**: Enrichment factor; **F1**: Acid soluble; **F2**: Reducible; **F3**: Oxidizable fraction; **F4**: Residual.

**Table 2:** Average Cd concentration in the current study (mg kg^−1^) compared to the average of worldwide rivers in sediments (mg kg^−1^).

River	Country	Cd	Reference
Present study	Egypt	0.16	Present study
Yangtze River	China	0.98	[72]
Buriganga River	Bangladesh	0.8	[73]
Ipojuca River	Brazil	0.16	[74]
Ghaghara River	India	0.28	[75]
Nile River	Egypt	0.06	[66]
World average		1.4	[68]
UCC		0.09	[54]
USEPA		0.61	[69]
UCC		0.5	[76]

**Table 3:** Results of the Pearson’s correlation analysis of Nile River sediments and water cadmium concentration with water parameters, Zr, Cd fractions (%), and grain size (%).

	Cd (mg kg^−1^)	Cd (mg L^−1^)	PH	TDS	ORP	Temp	F1	F2	F3	F4	Sand	Silt	Clay
Cd (mg kg^−1^)	1.00												
Cd (mg L^−1^)	0.45	1.00											
PH	−0.16	−0.35	1.00										
TDS	0.18	0.67	−0.62	1.00									
ORP	0.39	0.13	−0.02	0.07	1.00								
Temp.	0.04	0.69	−0.19	0.64	−0.13	1.00							
(F1)	0.50	0.22	−0.08	0.08	0.13	0.22	1.00						
(F2)	0.31	−0.19	0.06	−0.25	0.31	−0.25	0.60	1.00					
(F3)	0.10	0.12	−0.10	0.09	−0.08	0.23	0.28	0.47	1.00				
(F4)	−0.47	−0.12	0.06	0.01	−0.18	−0.11	−0.95	−0.81	−0.49	1.00			
Zr	−0.15	−0.35	0.19	−0.18	0.39	−0.20	−0.05	0.12	0.07	−0.01			
Sand	−0.32	−0.07	0.07	−0.17	−0.36	−0.02	−0.36	−0.54	−0.67	0.52	1.00		
Silt	0.34	0.08	−0.06	0.18	0.39	0.05	0.38	0.56	0.65	−0.54	−0.99	1.00	
Clay	0.06	−0.04	−0.08	0.03	0.00	−0.14	0.12	0.20	0.51	−0.22	−0.67	0.57	1.00

**ORP**: Oxidation Reduction Potential (mV), **TDS**: Total dissolved (mg kg^−1^), **Temp.**: Temperature (°C), **F1**: Acid soluble; **F2**: Reducible; **F3**: Oxidizable fraction; **F4**: Residual fraction.

**Table 4:** Correlation between the bioavailability fraction of Cd (F1 %) in this study with Worldwide rivers.

River	Country	Cd Fraction (%)	Fraction	Method	Reference
Ergen River	Turkey	25%	Acid soluble	BCR modification	[85]
Yamuna	India	(>70%)	Exchangeable + Carbonate	Tiesser et al., 1979	[86]
Xijing River	China	44.80%	Acid soluble	BCR modification	[87]
Gomti River	India	(17–28)%	Exchangeable + Carbonate	Tiesser et al., 1979	[88]
Odra River	Germany/poland	(23–39)%	Exchangeable + Carbonate	Tiesser et al., 1979	[89]
Odiel River	Spain	(15–70)%	Acid soluble	BCR modification	[90]
Present study	Egypt	27.11%	Acid soluble	BCR	

**Table 5:** Cadmium concentration in water (mg L^−1^) and water parameters of Nile River sediments (PH, TDS, ORP, Temp.).

Sample Location	Cd (mg/L)	PH	TDS	ORP	Temp (°C)
Giza	0.01	8	188	313	31.3
Cairo	0.009	7.96	186	352	30
Helwan	0.008	7.9	181	372	29.8
Naser	0.005	8.28	162	362	28.6
Beni Suef	0.007	8.61	157	372	28.7
Biba	0.002	8.44	149	352	28.5
Minya	0.003	8.54	151	435	28
Bni Mazar	0.005	8.66	158	441	28
Samalut	0.006	8.7	162	356	27.8
Asyut	0.005	8.43	153	331	27
Abu Tij	0.003	8.4	156	322	27.7
Sidaf	0.002	8.5	154	328	27.3
Girga	0.002	8.6	154	330	27.1
Sohag	0.007	8.43	150	391	27.5
Tahta	0.002	8.41	166	342	27.4
Nagaa Hammadi	0.001	8.65	158	352	28
Qena	0.006	9	152	335	29
Luxor	0.005	8.68	148	239	30
Armant	0.004	8.36	155	300	29.5°
Esna	0.002	8.37	157	340	26.5°
Edfu	0.003	8.43	151	308	26.1°
KomUmbu	0.001	8.3	146	290	26°
Aswan	0.004	8.1	149	392	26°
**Average**	0.004	8.42	158.39	345.87	8.42
**Maximum**	0.01	9	188	441	9
**Minimum**	0.001	7.90	146	239	7.9

**ORP:** Oxidation Reduction Potential (mV), **TDS**: Total dissolved solids (mg kg^−1^), **Temp.**: Temperature (°C).

## Data Availability

The data used to support the investigations of this study are included in the manuscript.

## Abbreviations

ICP-MS	Inductively Coupled Plasma Mass Spectrometry
ICP-AES	Inductively Coupled Plasma Atomic Emission Spectroscopy
TDS	Total Dissolved Solids.
ORP	Oxidation-Reduction Potential.
BCR	European Community Bureau of Reference
WHO	World Health Organization.
USEPA	United States Environmental Protection Agency.
CCME	Canadian Council of Ministers of Environment
ATSDR	Agency for Toxic Substances and Disease Registry, USA
